# Dexamethasone-sparing strategies in anthracycline and cyclophosphamide-based chemotherapy with a focus on 5-HT3 receptor antagonists: a network meta-analysis

**DOI:** 10.3389/fonc.2024.1414037

**Published:** 2024-07-26

**Authors:** Daichi Watanabe, Hirotoshi Iihara, Ryo Kobayashi, Hironori Fujii, Ryutaro Mori, Keisuke Kumada, Masahito Shimizu, Manabu Futamura, Akio Suzuki

**Affiliations:** ^1^ Department of Pharmacy, Gifu University Hospital, Gifu, Japan; ^2^ Innovative and Clinical Research Promotion Center, Gifu University Hospital, Gifu, Japan; ^3^ Patient Safety Division, Gifu University Hospital, Gifu, Japan; ^4^ Laboratory of Pharmacy Practice and Social Science, Gifu Pharmaceutical University, Gifu, Japan; ^5^ Laboratory of Advanced Medical Pharmacy, Gifu Pharmaceutical University, Gifu, Japan; ^6^ Department of Breast Surgery, Gifu University Hospital, Gifu, Japan; ^7^ Department of Emergency and Disaster Medicine, Gifu University Graduate School of Medicine, Gifu, Japan; ^8^ Department of Gastroenterology, Gifu University Graduate School of Medicine, Gifu, Japan

**Keywords:** antiemetics, neurokinin-1 receptor antagonists, serotonin 5-HT3 receptor antagonists, dexamethasone, nausea, vomiting, anthracyclines, cyclophosphamide

## Abstract

**Background:**

The effectiveness of a dexamethasone-sparing strategy in the treatment of breast cancer with anthracycline-cyclophosphamide therapy when combined with first-generation 5-HT3 receptor antagonists (RAs) and neurokinin-1 RAs is unclear. This is attributable to a lack of evidence from direct comparison of multiple doses of DEX to a single dose of DEX in combination with first-generation 5-HT3 RAs in anthracycline-cyclophosphamide therapy. Our goal was to clarify the impact of dexamethasone-sparing strategies that involve both first-generation 5-HT3 RAs and palonosetron when combined with neurokinin-1 RAs, using a network meta-analysis.

**Materials and methods:**

A literature search was conducted on PubMed/Medline for articles published up to July 4, 2023. We included randomized controlled trials which assessed the efficacy of antiemetic regimens which combined 5-HT3 RAs and dexamethasone, with or without neurokinin-1 RAs, for the initial dose in anthracycline-cyclophosphamide therapy for patients with breast cancer. The primary outcome was the proportion of patients achieving a complete response during the delayed phase (CR-DP).

**Results:**

The difference in the proportion of patients achieving CR-DP between multiple and single doses of dexamethasone was 0.1% (95%CI: -12.4 to 12.5) with palonosetron and neurokinin-1 RAs, compared to 5.3% (95%CI: -13.4 to 23.0) with a single dose of a first-generation 5-HT3 receptor antagonist. Additionally, the difference was 12.7% (95% CI: -2.8 to 28.2) when comparing palonosetron against first-generation 5-HT3 RAs in combination with a single dose of dexamethasone and neurokinin-1 RAs.

**Conclusion:**

Palonosetron is recommended rather than a single dose of first-generation 5-HT3 RAs in dexamethasone-sparing strategies for anthracycline-cyclophosphamide therapy.

## Introduction

Chemotherapy-induced nausea and vomiting (CINV) greatly affects patients’ quality of life, treatment adherence, and therapy effectiveness, and ranks as the second-most aversion condition after death among those receiving chemotherapy (1). Reducing CINV is crucial for improving patient well-being and maintaining chemotherapy continuity.

Anthracycline-cyclophosphamide (AC) therapy, commonly used for breast cancer, often causes severe nausea and vomiting and is classified as highly emetogenic. A triplet antiemetic regimen of dexamethasone (DEX), 5-HT3 receptor antagonists (5HT3 RAs), and neurokinin-1 receptor antagonists (NK1 RAs) effectively reduces CINV, as evidenced by several phase III studies (2–8), and is recommended in global guidelines (9, 10).

Palonosetron is a second-generation 5HT3 RA that has a longer half-life and higher binding affinity compared to first-generation 5HT3 RAs (1st 5HT3 RAs) (11, 12). Palonosetron has been demonstrated to be more effective at preventing CINV than 1st 5HT3 RAs in patients receiving AC therapy and moderately emetogenic chemotherapy (13–15). It allows the DEX-sparing strategy, which limits dosing to a single dose of DEX rather than multiple doses to minimize corticosteroids side effects (16–18). The DEX-sparing strategy alongside palonosetron has been shown to be as effective as multiple DEX doses in the prophylaxis of CINV (19–25). In AC therapy, the DEX-sparing strategy in combination with palonosetron and an NK1 RA demonstrated acceptable differences in the complete response rate during both the overall phase (-1.1; 95% CI: -12.0 to 9.8) and the delayed phase (-3.3; 95% CI: -14.4 to 7.8) (26). Consequently, the DEX-sparing strategy has become widely adopted in AC therapy (9, 27).

Even though the DEX-sparing strategy has been established in combination with palonosetron, global guidelines have not specified the type of 5HT3 RA to be used for DEX-sparing in AC therapy (9, 10, 27). Using DEX-sparing with 1st 5HT3 RAs might be inadequate in preventing delayed-onset CINV associated with AC therapy, as symptoms persist beyond the first day (28).

The impact of the DEX-sparing strategy with 1st 5HT3 RAs on AC therapy is unknown. This network meta-analysis aimed to compare the efficacy of a single dose of DEX with that of multiple doses of DEX when combined with 1st 5HT3 RAs based on data from randomized control trials.

## Materials and methods

### Search strategy and eligibility criteria

We included randomized controlled trials which evaluated the effectiveness of antiemetic regimens that combined 5HT3RA and DEX, with or without NK1RA, for the initial dose in AC-based regimens for patients with breast cancer. Crossover studies were eligible only if they offered data for the first cycle. If over 5% of participants were given a non-AC regimen, studies were included only if they presented outcome data specifically for patients who received AC therapy, such as through subgroup analysis. Our selection was limited to studies written in English.

We classified each antiemetic regimen based on the duration of DEX administration, the type and duration of 5HT3 RA used, and concomitant NK1 RA. We divided DEX into two categories: a single dose of DEX, given only on the first day, and multiple doses of DEX, administered from the second day onwards. Similarly, we categorized 5HT3 RA into three groups: a single dose of a 1st generation 5HT3 RA, comprising ondansetron, granisetron, or ramosetron, administered on the first day only; multiple doses of 1st 5HT3 RA, given from the second day onwards; and palonosetron. NK1 RAs, including aprepitant, fosaprepitant, casopitant, rolapitant, netupitant, and fosnetupitant, were regarded as equivalent. Different dosages or routes of administration of the same agent were regarded as equivalent. An overview of all experimental antiemetic regimens included in our analysis is available in **Supplementary Table 1**.

This study followed the Preferred Reporting Items for Systematic Review and Meta-Analyses (PRISMA) extension statement for the reporting of systematic reviews incorporating network meta-analyses (29, 30). The protocol for this review was previously published in the PROSPERO database (CRD42024511693).

### Information sources

We conducted a systematic search for eligible studies published up to July 4, 2023, utilizing PubMed/Medline databases. Additionally, we performed a manual search through the reference lists of pertinent reviews and meta-analyses. The specific search terms employed are detailed in **Supplementary Table 2**.

### Study selection and data extraction

Two reviewers (DW and HI) independently screened all relevant studies in duplicate to confirm their eligibility and extracted the following information: author, title, publication source, date of publication, and language; factors contributing to risk of bias evaluation (randomization methods, allocation concealment, blinding method, handling of incomplete outcome data, selective outcome reporting, and identification of other potential biases); study characteristics (trial design, participant source, inclusion and exclusion criteria, subgroup analyses, adherence to allocated intervention); participant details (demographics such as age and sex/gender, total number recruited, allocated, and assessed, cancer type, antineoplastic treatments, known CINV risk factors); intervention and comparator specifics (antiemetic agents, dosages, prophylaxis duration); and outcomes. All outcome measures were extracted from the first planned chemotherapy cycle. Discrepancies between reviewers were resolved through discussion with a third author (RK) to achieve consensus.

### Outcome measures

The primary outcome was a complete response during the delayed phase (CR-DP), defined as the absence of emesis and no use of rescue medication from 24 to 120 hours post-chemotherapy initiation. The results were reported as the proportion of patients achieving CR-DP. For comparisons between antiemetic regimens, both the risk difference and risk ratio are presented.

### Risk of bias assessment

Two reviewers (DW and HI) independently assessed the risk of bias due to the randomization process, deviations from the intended interventions, missing outcome data, measurement of the outcome, selection of the reported results, and other biases of the included studies using the Revised Cochrane risk-of-bias tool for randomized trials (RoB 2) by The Cochrane Collaboration (http://www.cochrane.de). Any disagreements were resolved through discussion with a third reviewer (RK) to achieve consensus.

### Statistical analysis

An arm-based network meta-analysis using Bayesian methods was conducted to compare the CR-DP rates of multiple antiemetic strategies. Network meta-analysis enables both the direct comparison of treatments in individual trials and indirect comparison across trials (31). Notably, an arm-based approach estimates population-averaged, treatment-specific event rates. The CR-DP rate for each antiemetic regimen was aggregated using the nma.ab.bin function within the R package pcnetmeta (32). This model accounts for heterogeneity in the variance of random effects and the correlation between different treatments within each cohort. Final estimation routines involved 3 chains, each with 50,000 burn-in iterations, followed by 100,000 estimation iterations without thinning, resulting in a total of 150,000 iterations for analysis. The results of the network meta-analysis were reported as the posterior median with corresponding 95% credible intervals (CIs). Statistical significance was assessed using 95% CIs. We also performed standard random effects and fixed effect meta-analysis to aggregate the proportion of patients achieving CR-DP with each antiemetic regimen using the metaprop function in the R package meta (33, 34). All analyses were performed in R, version 4.3.2.

## Results

### Eligible studies and characteristics

**Figure 1:**
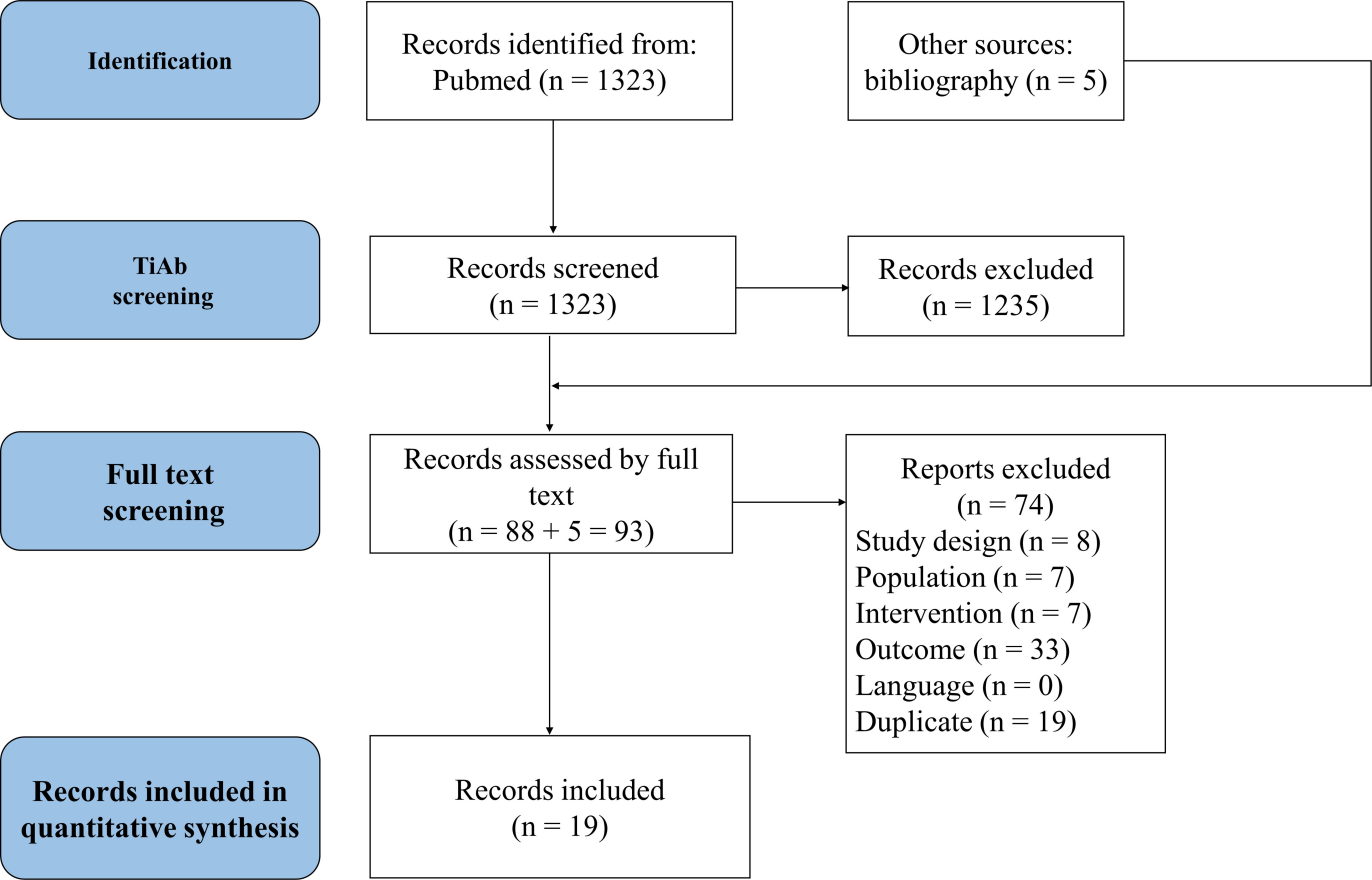
Study selection.

Through literature research, we identified 1,323 potentially relevant references. After reviewing titles and abstracts, 88 studies were selected. An additional 5 studies were included via manual search, resulting in 93 studies undergoing full-text review. Ultimately, 21 studies met our inclusion criteria; however, two were excluded from analysis due to the absence of CR-DP data. These exclusions included a study on patients who had previously undergone chemotherapy (2) and another which compared fosnetupitant with fosaprepitant (35). As a result, 19 studies were included in our network meta-analysis, as depicted in the Prisma flow diagram in **Figure 1**.

**Supplementary Table 3** presents the key characteristics of the included studies, with antiemetic regimens organized into 10 categories by the antiemetic agents used. **Supplementary Table 4** offers detailed information on the agents included in each antiemetic regimen. Risk of bias tables for the included studies are shown in **Supplementary Figures 1** and **2**.

A total of 9,108 patients across 19 studies were included in our network meta-analysis (3–8, 21, 23, 26, 36–45). These studies were published between 2005 and 2021, of which 14 (73.6%) were double-blind randomized controlled trials (3–8, 26, 36, 38–42, 45), and sample size ranged from 40 to 1,917 patients. Almost all participants were women with breast cancer who were naïve to emetogenic chemotherapy and received an AC-based regimen.

To summarize treatments involving 6,187 patients with NK1 RA, 51.7% received aprepitant/fosaprepitant, 23.2% casopitant, 19.5% netupitant, and 5.6% rolapitant. Regarding the administration of DEX, among 2,158 patients given multiple doses of DEX, 78.0% took 8 mg and 22.0% took 4 mg. Additionally, 61.5% received 3-day doses and 38.5% received 4-day doses. Regarding the administration of palonosetron, among 3,475 patients given palonosetron, 28.8% received 0.75 mg, 51.5% received 0.5 mg, 19.0% received 0.25 mg, and 0.7% received 0.075 mg.

**Figure 2:**
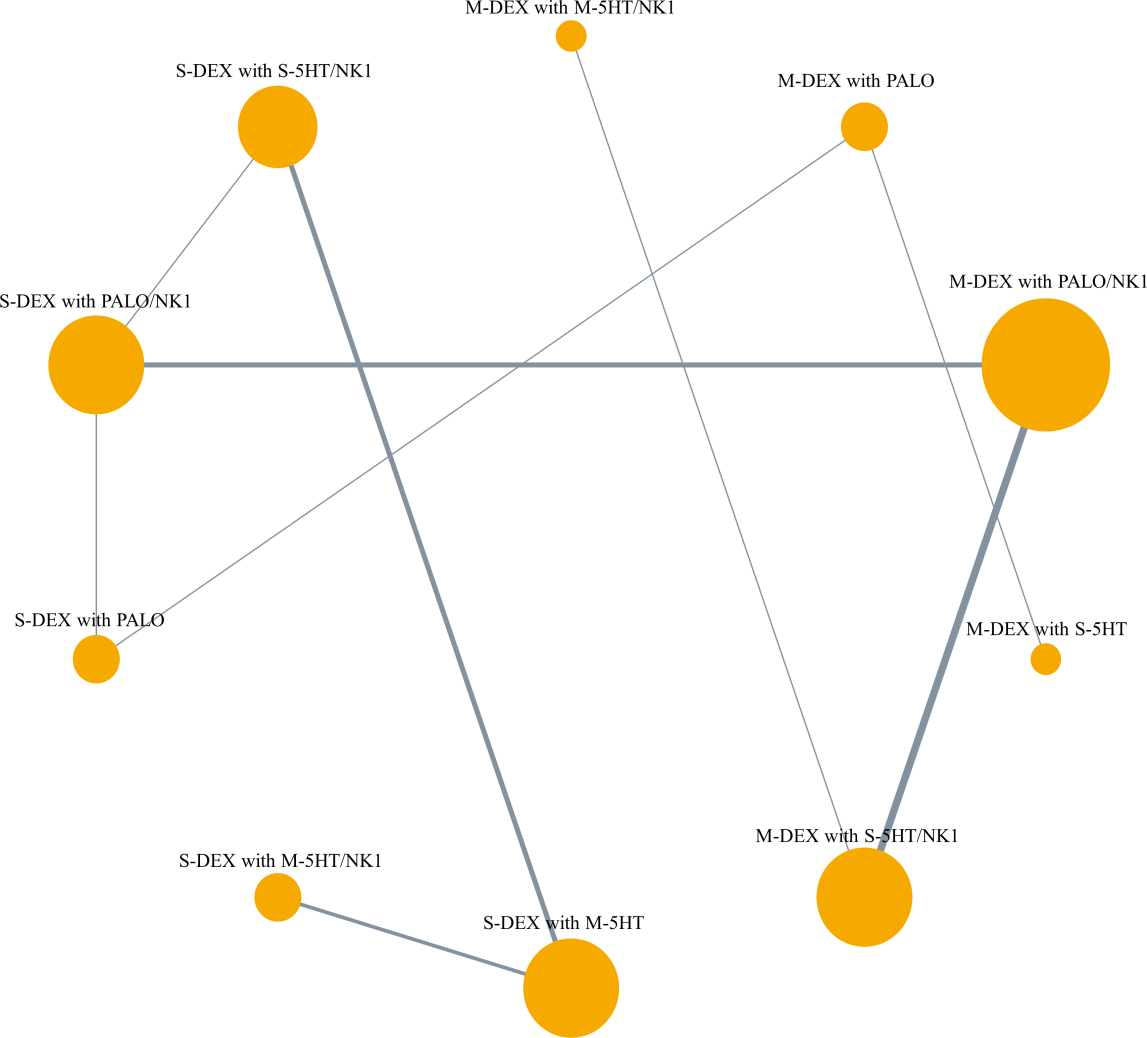
Network plot of primary outcome, complete response during the delayed phase. The lines represent direct comparisons between treatments in trials. Line thickness indicates the number of trials evaluated in each comparison. Node size indicates the number of participants assigned to each treatment. M-DEX with M-5HT/NK1, multiple doses of dexamethasone in combination with multiple doses of a first-generation 5-HT3 receptor antagonist and neurokinin-1 receptor antagonist; M-DEX with PALO, multiple doses of dexamethasone in combination with palonosetron; M-DEX with PALO/NK1, multiple doses of dexamethasone in combination with palonosetron and neurokinin-1 receptor antagonist; M-DEX with S-5HT, multiple doses of dexamethasone in combination with a single dose of a first-generation 5-HT3 receptor antagonist; M-DEX with S-5HT/NK1, multiple doses of dexamethasone in combination with a single dose of a first-generation 5-HT3 receptor antagonist and neurokinin-1 receptor antagonist; S-DEX with M-5HT, single dose of dexamethasone in combination with multiple doses of a first-generation 5-HT3 receptor antagonist; S-DEX with M-5HT/NK1, single dose of dexamethasone in combination with multiple doses of a first-generation 5-HT3 receptor antagonist and neurokinin-1 receptor antagonist; S-DEX with PALO, single dose of dexamethasone in combination with palonosetron; S-DEX with PALO/NK1, single dose of dexamethasone in combination with palonosetron and neurokinin-1 receptor antagonist; S-DEX with S-5HT/NK1, single dose of dexamethasone in combination with a single dose of a first-generation 5-HT3 receptor antagonist and neurokinin-1 receptor antagonist.

The antiemetic regimens that were directly compared for CR-DP in each study were as follows: Four studies compared multiple doses of DEX with a single dose, in combination with palonosetron (21, 23, 26, 38). Three of these studies also used a NK1RA (23, 26, 38). Six studies compared the use of NK1RA with its absence, in combination with DEX and 5HT3 RA (3–8). Six studies compared palonosetron with 1st 5HT3 RAs (36, 37, 39, 43–45). Additionally, three studies compared different dosages or methods of administering 5HT3 RAs (40–42). Notably, none of the studies directly compared multiple doses of DEX with a single dose when combined with a 1st 5HT3 RA and NK1RA. The network plot of CR-DP is shown in **Figure 2**.

### Complete response rate during the delayed phase in arm-based network meta-analysis

Our primary outcome, CR-DP rate, is presented for each eligible study in **Supplementary Table 5**, and the pooled CR-DP rate at the cohort level (by classified antiemetic regimen) in the arm-based network meta-analysis is presented in **Table 1**. Cohorts with multiple or single doses of DEX combined with palonosetron and NK1 RA accounted for seven and six studies, respectively. Both groups showed a CR-DP rate of 72.3% (95%CI: 61.2 to 81.1) for multiple and 72.3% (95%CI: 60.4 to 81.0) for single doses of DEX. When combined with single doses of 1st 5HT3 RA and NK1 RA, the studies included five for multiple and four for single doses of DEX, respectively. CR-DP rates were 64.7% (95%CI: 50.5 to 76.3) for multiple and 59.3% (95%CI: 46.3 to 71.0) for single doses of DEX. When combined with multiple doses of 1st 5HT3 RA and NK1 RA, only a few studies were available: one for multiple and two for single doses of DEX. CR-DP rates were 62.6% (95%CI: 34.2 to 85.0) for multiple and 69.0% (95%CI: 49.9 to 84.3) for single doses of DEX. The results of aggregated proportion and heterogeneity of CR-DP in each antiemetic regimen using meta-analysis of proportions are shown in **Supplementary Table 6**.

**Table 1:** Pooled proportions of patients achieving complete response during the delayed phase for each antiemetic regimen using arm-based network meta-analysis.

Antiemetic regimen			Cohort using the regimen (number of studies)	Complete response during delayed phase /patients using regimen	Pooled complete response rate, median (95% credible interval)
DEX dose	5HT3 RA	NK1 RA			
Multiple- day	Palonosetron	Use	7 (7 study)	531/744	72.3% (61.2, 81.1)
Single- day	Palonosetron	Use	7 (6 study)	1346/1844	72.3% (60.4, 81.0)
Multiple- day	Single dose of 1st 5HT3 RA	Use	5 (5 study)	363/587	64.7% (50.5, 76.3)
Single- day	Single dose of 1st 5HT3 RA	Use	4 (4 study)	539/939	59.3% (46.3, 71.0)
Multiple- day	Multiple doses of 1st 5HT3 RA	Use	1 (1 study)	185/291	62.6% (34.2, 85.0)
Single- day	Multiple doses of 1st 5HT3 RA	Use	4 (2 study)	1283/1782	69.0% (49.9, 84.3)
Multiple- day	Palonosetron	Absent	2 (2 study)	192/300	67.0% (45.6, 82.8)
Single- day	Palonosetron	Absent	5 (3 study)	573/857	59.9% (41.8, 74.2)
Multiple- day	Single dose of 1st 5HT3 RA	Absent	1 (1 study)	118/236	49.5% (21.6–76.5)
Single- day	Multiple doses of 1st 5HT3 RA	Absent	5 (5 study)	848/1528	55.6% (44.5, 66.0)

1st 5HT3 RA, first-generation 5-HT3 receptor antagonist; 5HT3 RA, 5-HT3 receptor antagonist; DEX, dexamethasone; NK1 RA, neurokinin-1 receptor antagonist.

**Figure 3:**
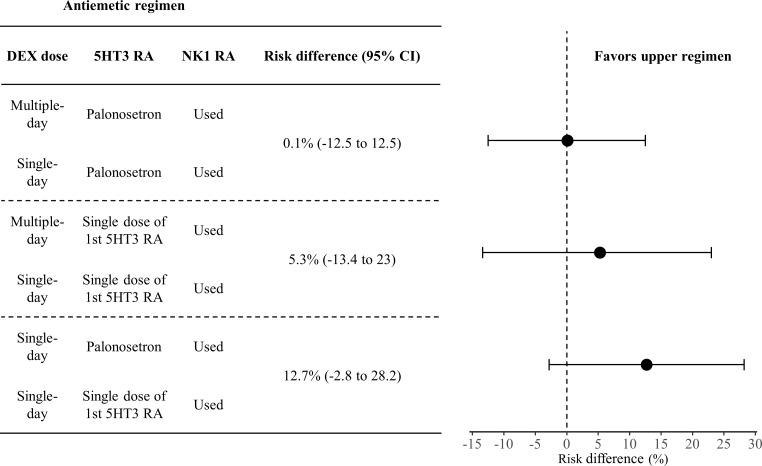
Forest plot of risk difference in complete response during the delayed phase. Effect sizes are from the network meta-analysis.

**Figure 4:**
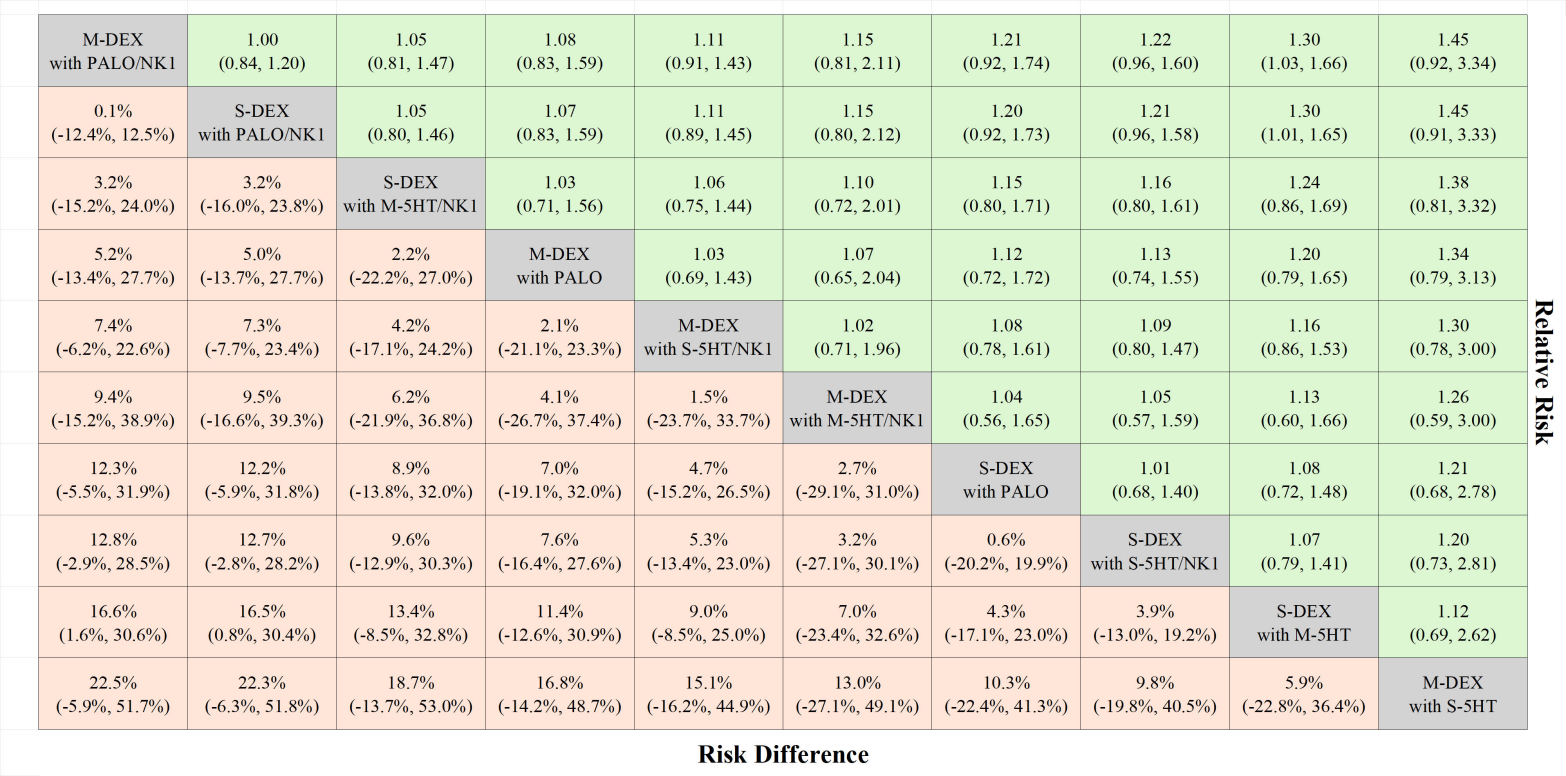
League table complete responses during the delayed phase. The treatments are listed according to the highest complete response rate during the delayed phase. Each cell presents the median risk difference and its associated 95% credible interval for comparison (treatment in the column versus treatment in the row), along with the median risk ratio and its 95% credible interval for reverse comparison (treatment in the row versus treatment in the column). M-DEX with M-5HT/NK1, multiple doses of dexamethasone in combination with multiple doses of a first-generation 5-HT3 receptor antagonist and neurokinin-1 receptor antagonist; M-DEX with PALO, multiple doses of dexamethasone in combination with palonosetron; M-DEX with PALO/NK1, multiple doses of dexamethasone in combination with palonosetron and neurokinin-1 receptor antagonist; M-DEX with S-5HT, multiple doses of dexamethasone in combination with a single dose of a first-generation 5-HT3 receptor antagonist; M-DEX with S-5HT/NK1, multiple doses of dexamethasone in combination with a single dose of a first-generation 5-HT3 receptor antagonist and neurokinin-1 receptor antagonist; S-DEX with M-5HT, single dose of dexamethasone in combination with multiple doses of a first-generation 5-HT3 receptor antagonist; S-DEX with M-5HT/NK1, single dose of dexamethasone in combination with multiple doses of a first-generation 5-HT3 receptor antagonist and neurokinin-1 receptor antagonist; S-DEX with PALO, single dose of dexamethasone in combination with palonosetron; S-DEX with PALO/NK1, single dose of dexamethasone in combination with palonosetron and neurokinin-1 receptor antagonist; S-DEX with S-5HT/NK1, single dose of dexamethasone in combination with a single dose of a first-generation 5-HT3 receptor antagonist and neurokinin-1 receptor antagonist.

**Figure 3** shows the risk differences in complete response during the delayed phase. The difference for DEX in multiple versus single doses, when used with palonosetron and NK1 RA, was 0.1% (95% CI: -12.4 to 12.5). In contrast, this difference increased to 5.3% (95% CI: -13.4 to 23.0) on comparison of DEX doses with 1st 5HT3 RA and NK1 RA, and additionally 12.7% (95% CI: -2.8 to 28.2) when comparing palonosetron against 1st 5HT3 RAs with a single dose of DEX or NK1 RA. All pairwise comparisons are shown in **Figure 4**.

## Discussion

Our network meta-analysis showed a minimal difference of 0.1% in CR-DP rates with a DEX-sparing regimen combined with palonosetron and NK1 RA. This finding supports previous evidence (23, 26, 38). However, this gap widened to 5.3% when DEX-sparing was combined with 1st 5HT3 RA and NK1 RA. Additionally, the difference was 12.7% (95% CI: -2.8 to 28.2) on comparison of palonosetron against 1st 5HT3 RAs with a single dose of DEX and NK1 RA, marking a clinically meaningful difference of 10%, as deemed by the MASCC/ESMO guideline (46). Based on these findings, the concurrent use of palonosetron is recommended.

CINV related to AC therapy persists beyond the first day (28). Effective prevention in the initial cycle is vital for subsequent cycle management success. Conversely, a prolonged CINV duration correlates with minimal improvement in subsequent cycles (47), highlighting the importance of early CINV control. For managing delayed-phase CINV, palonosetron has shown a better control rate than 1st 5HT3 receptor antagonists when combined with DEX and NK1 RAs (36, 37, 43, 45). Additionally, a meta-analysis comparing 1st 5HT3 RAs with palonosetron revealed significant benefits associated with palonosetron (15). Our present results also indicate an improved CR-DP rate with palonosetron usage compared to a single dose of 5HT3 RA. Consequently, incorporating palonosetron from the first cycle of AC therapy is advisable for comprehensive CINV management throughout the entire course of AC therapy.

This study did not distinguish between the administration of different dosages of the same antiemetics. Regarding DEX dosage, the complete protection ratio for both the acute and delayed phases were equivalent in comparing 24 mg and 8 mg doses on day 1 (48). On the other hand, in the delayed phase, no confirmatory trial has compared different DEX dosages. However, in our network meta-analysis, all patients who received a triplet antiemetic regimen of DEX, 5HT3 RAs, and NK1 RAs took 8 mg of DEX. Regarding palonosetron dosage, several phase III trials have shown that palonosetron 0.75 mg is as effective as 0.25 mg, suggesting that the 0.25 mg dose is sufficient to achieve efficacy (13, 14, 49). However, palonosetron 0.75 mg is predominantly used in some countries like Japan. This preference is based on a phase III trial conducted in Japan (39), which demonstrated that palonosetron 0.75 mg was superior to 1st 5HT3 RAs in achieving a higher CR-DP rate for highly emetogenic chemotherapy. Our network meta-analysis included patients receiving palonosetron doses of 0.75 mg, 0.5 mg, and 0.25 mg, comprising 28.8%, 51.5%, and 19.0% of the total population, respectively. This distribution indicates that the analysis was not heavily weighted towards any particular dose within the 0.25–0.75 mg range. Therefore, it is reasonable to regard the different dosages of these antiemetics as equivalent.

The strength of this study stems from its strict inclusion of randomized controlled trials, which ensures a robust evidence base. Further, our network meta-analysis was limited to studies of patients with breast cancer undergoing AC therapy, ensuring consistency across key CINV risk factors such as chemotherapy regimen, patient age, sex, prior chemotherapy history, and dexamethasone dosages. This uniformity across trials facilitates the integration of various antiemetic regimens in a network meta-analysis. Additionally, we ascertained treatment-specific CR-DP rates and their differences across a range of antiemetic regimens through an arm-based network meta-analysis. Previous studies that compared different antiemetic regimens through contrast-based network meta-analyses (50–52) have commonly reported odds ratios only, potentially creating unnecessary obstacles for patients and clinicians in fully understanding and evaluating the efficacy of antiemetic treatments (31, 53–55).

We also acknowledge several limitations. First, comparisons between multiple doses and a single dose of DEX combined with 1st 5-HT3 RAs are based mainly on indirect comparisons, which cannot replace the direct comparisons obtained from randomized studies.

Second, the scope of our network meta-analysis was confined to the examination of antiemetic strategies, specifically those involving DEX, 5HT3 RAs, and NK1 RAs. Regarding olanzapine’s use for preventing CINV in AC therapy, our preliminary survey identified no randomized controlled trials of the efficacy of a DEX-sparing strategy for patients undergoing AC therapy. The number of trials that included olanzapine in at least one treatment arm was also limited (56–58). Other studies have noted concerns about undefined classification of 5-HT3 receptor antagonists (59) and a study design which was restricted to patients at high risk of CINV (60). Therefore, even if a network meta-analysis is performed, only an incomplete network can be formed, and it is not appropriate to indirectly compare the efficacy of DEX-sparing in olanzapine-combination regimens.

Third, our network meta-analysis lacks direct comparison of multiple versus single DEX doses in combination with multiple doses of 1st 5-HT3 RAs. Furthermore, only a few studies have incorporated the combination of multiple doses of 1st 5-HT3 RAs with DEX and NK1 RAs. Consequently, this has resulted in broad credible intervals for our estimates of the proportion of patients achieving CR-DP and its difference, which means in turn that we lack sufficient evidence to recommend a DEX-sparing approach combined with multiple doses of 1st 5-HT3 RAs.

Finally, our study’s evaluation focused exclusively on CR-DP outcomes. Other outcomes, such as the absence of nausea, did not allow the establishment of connections within the treatment network comparing multiple doses to a single dose of DEX when combined with 1st 5-HT3 RAs and NK1 RAs in the network meta-analysis.

## Conclusions

For patients with breast cancer undergoing AC therapy, a DEX-sparing strategy that involves use of a single dose of a 1st 5-HT3 receptor antagonist is suggested to be inadequate. Consequently, based on current evidence, palonosetron is the preferred option.

## Data availability statement

The original contributions presented in the study are included in the article/**Supplementary Material**. Further inquiries can be directed to the corresponding author.

## Author contributions

DW: Conceptualization, Formal analysis, Methodology, Visualization, Writing – original draft. HI: Conceptualization, Supervision, Writing – review & editing. RK: Supervision, Writing – review & editing. HF: Supervision, Writing – review & editing. RM: Supervision, Writing – review & editing. KK: Supervision, Writing – review & editing. MS: Supervision, Writing – review & editing. MF: Supervision, Writing – review & editing. AS: Conceptualization, Supervision, Writing – review & editing.
