# Supplementary material for: Dexamethasone-sparing strategies in anthracycline and cyclophosphamide-based chemotherapy with a focus on 5-HT3 receptor antagonists: a network meta-analysis
Source: Front Oncol. 2024 Jul 26;14:1414037. doi: 10.3389/fonc.2024.1414037 (PMC11310115; doi:10.3389/fonc.2024.1414037)
Supplement: Supplementary file 1 [file DataSheet_1.pdf]

Supplementary material

Supplementary Table 1: Overview of all antiemetic regimens in network meta-analysis 2
Supplementary Table 2: Search terms 3
Supplementary Table 3: Characteristics of eligible studies. 4
Supplementary Table 4: Antiemetic regimens each study included in network meta-analysis 9
Supplementary Figure 1: Risk of bias summary: review authors' judgements about each risk of bias item for each included study. 12
Supplementary Figure 2: Risk of bias graph 13
Supplementary Table 5: Proportion of patients achieving a complete response during the delayed phase in eligible studies 14
Supplementary Table 6: Pooled proportion of patients achieving a complete response during the delayed phase for each antiemetic regimen using random- and fixed-effect models 16

Supplementary Table 1: Overview of all antiemetic regimens in network meta-analysis

Acronym	Antiemetic regimen
M-DEX with PALO/NK1	Multiple doses of dexamethasone in combination with palonosetron and neurokinin-1 receptor antagonist.
M-DEX with M-5HT/NK1	Multiple doses of dexamethasone in combination with multiple doses of a first-generation 5-hydroxytryptamine3 receptor antagonist and neurokinin-1 receptor antagonist.
M-DEX with S-5HT/NK1	Multiple doses of dexamethasone in combination with a single dose of a first-generation 5-hydroxytryptamine3 receptor antagonist and neurokinin-1 receptor antagonist.
S-DEX with PALO/NK1	Single dose of dexamethasone in combination with palonosetron and neurokinin-1 receptor antagonist.
S-DEX with M-5HT/NK1	Single dose of dexamethasone in combination with multiple doses of a first-generation 5-hydroxytryptamine3 receptor antagonist and neurokinin-1 receptor antagonist.
S-DEX with S-5HT/NK1	Single dose of dexamethasone in combination with a single dose of a first-generation 5-hydroxytryptamine3 receptor antagonist and neurokinin-1 receptor antagonist.
M-DEX with PALO	Multiple doses of dexamethasone in combination with palonosetron.
M-DEX with S-5HT	Multiple doses of dexamethasone in combination with a single dose of a first-generation 5-hydroxytryptamine3 receptor antagonist.
S-DEX with PALO	Single dose of dexamethasone in combination with palonosetron.
S-DEX with M-5HT	Single dose of dexamethasone in combination with multiple doses of a first-generation 5-hydroxytryptamine3 receptor antagonist.

Supplementary Table 2: Search terms

("nausea"[TW] OR "vomit*"[TW] OR "nausea or vomiting"[TW] OR "nausea and vomiting"[TW] OR "cinv"[TW] OR "emesis"[TW] OR "complete control"[TW] OR "total control"[TW] OR "complete protection"[TW])
AND
("doxorubicin"[TW] OR "adriamycin"[TW] OR "epirubicin"[TW] OR "anthracycline*"[TW] OR "cyclophosphamide"[TW] OR "dose dense AC"[TW] OR "dose-dense AC"[TW] OR "ddAC"[TW] OR "AC regimen"[TW] OR "AC therapy"[TW] OR "AC chemotherapy"[TW] OR "EC regimen"[TW] OR "EC therapy"[TW] OR "EC chemotherapy"[TW] OR "FEC regimen"[TW] OR "FEC therapy"[TW] OR "FEC chemotherapy"[TW] OR "CAF regimen"[TW] OR "CAF therapy"[TW] OR "CAF chemotherapy"[TW])
AND
("Breast Neoplasms"[mh] OR "Breast*"[TW] OR "high emet*"[TW] OR "highly emet*"[TW] OR "high-emet*"[TW] OR "highly-emet*"[TW] OR "HEC"[TW] OR "moderate emet*"[TW] OR "moderately emet*"[TW] OR "moderate-emet*"[TW] OR "moderately-emet*"[TW] OR "MEC"[TW])

Supplementary Table 3: Characteristics of eligible studies.

Study (PMID)	Publication date	Blinding	Acronym	Patients (n)[*1]	Age (median)	Sex (%, female)	Naive to emetogenic chemotherapy (%)	Breast cancer (%)	AC-based regimens (%)[*2]
Aapro et al (24603643)	2014	double-blind	S-DEX with PALO	725	54	97.9	100	97.2	100
			S-DEX with PALO/NK1	724	54	98.2	100	97.7	100
Celio et al (20574663)	2012	open-label	S-DEX with PALO	52	NA	NA	NA	NA	100
			M-DEX with PALO	61	NA	NA	NA	NA	100
Herrstedt et al (19805683)	2009	double-blind	S-DEX with M-5HT	479	52	98	100	95	100
			S-DEX with M-5HT/NK1	479	51	98	100	96	100
			S-DEX with M-5HT/NK1	480	51	98	100	97	100
			S-DEX with M-5HT/NK1	479	53	98	100	95	100

Ito et al (29443652)	2018	double-blind	S-DEX with PALO/NK1	155	54.1	NA	NA	NA	100
			M-DEX with PALO/NK1	151	55	NA	NA	NA	100
Kosaka et al (26349772)	2016	single-blind	S-DEX with PALO/NK1	39	52.6 (mean)	100	100	100	100
			M-DEX with PALO/NK1	41	53.5 (mean)	100	100	100	100
Matsumoto et al (32168551)	2020	double-blind	M-DEX with S-5HT/NK1	164	54	100	100	100	100
			M-DEX with PALO/NK1	162	54	100	100	100	100
Ogata et al (Not applicable)	2017	double-blind	S-DEX with S-5HT/NK1	245	NA	100	NA	100	100
			S-DEX with PALO/NK1	246	NA	100	NA	100	100
Ohzawa et al (25435944)	2015	open-label	M-DEX with S-5HT/NK1	21	53	100	NA	100	100
			M-DEX with PALO/NK1	19	53	100	NA	100	100
	2010	double-blind	S-DEX with M-5HT	204	NA	NA	100	NA	100

Rapoport et al (19568773)			S-DEX with S-5HT/NK1	199	NA	NA	100	NA	100
Roila et al (24323030)	2014	double-blind	S-DEX with PALO/NK1	278	50	100	100	100	99.9
			M-DEX with PALO/NK1	273	50	99.9	100	100	100
Saito et al (19135415)	2009	double-blind	M-DEX with S-5HT	236	NA	NA	NA	100	100
			M-DEX with PALO	239	NA	NA	NA	100	100
Schnadig et al (28579832)	2017	double-blind	M-DEX with S-5HT/NK1	298	53.8 (mean)	98.3	NA	100	100
			M-DEX with M-5HT/NK1	291	54.1 (mean)	99.3	NA	100	100
Schwartzberg et al (26272768)	2015	double-blind	S-DEX with M-5HT	359	NA	NA	NA	NA	100
			S-DEX with M-5HT/NK1	344	NA	NA	NA	NA	100
Schwartzberg et al (32162813)	2020	double-blind	S-DEX with PALO/NK1	200	55.6 (mean)	100	100	100	100
			S-DEX with PALO/NK1	202	55.2 (mean)	100	100	100	100

Segawa et al (19605507)	2009	double-blind	S-DEX with PALO	26	NA	NA	100	NA	100
			S-DEX with PALO	27	NA	NA	100	NA	100
			S-DEX with PALO	27	NA	NA	100	NA	100
Warr et al (15837996)	2005	double-blind	S-DEX with M-5HT	424	52.1 (mean)	100	100	100	99.1
			S-DEX with S-5HT/NK1	433	53.1 (mean)	99.5	100	100	98.6
Wenzell et al (23748485)	2013	open-label	M-DEX with S-5HT/NK1	20	52.9 (mean)	100	100	95	95
			M-DEX with PALO/NK1	20	50.9 (mean)	100	100	100	100
Yeo et al (18327706)	2009	double-blind	S-DEX with M-5HT	62	48.5	100	100	100	100
			S-DEX with S-5HT/NK1	62	46.5	100	100	100	100
Zelek et al (34216177)	2021	open-label	M-DEX with PALO/NK1	78	56 (mean)	97.4	100	93.6	96.2
			M-DEX with S-5HT/NK1	84	54 (mean)	95.2	100	96.4	96.4

Matsuura et al (35045185)	2022	double-blind	S-DEX with PALO/NK1	52	56	100	NA	100	100
			S-DEX with PALO/NK1	50	56	100	NA	100	100
Herrstedt et al (16104039)	2005	double-blind	S-DEX with M-5HT	359	NA	100	0	100	99.2
			S-DEX with S-5HT/NK1	385	NA	99.5	0	100	98.4

Abbreviations: M-DEX with M-5HT/NK1, multiple doses of dexamethasone in combination with multiple doses of a first-generation 5-hydroxytryptamine3 receptor antagonist and neurokinin-1 receptor antagonist; M-DEX with PALO, multiple doses of dexamethasone in combination with palonosetron; M-DEX with PALO/NK1, multiple doses of dexamethasone in combination with palonosetron and neurokinin-1 receptor antagonist; M-DEX with S-5HT, multiple doses of dexamethasone in combination with a single dose of a first-generation 5-hydroxytryptamine3 receptor antagonist; M-DEX with S-5HT/NK1, multiple doses of dexamethasone in combination with a single dose of a first-generation 5-hydroxytryptamine3 receptor antagonist and neurokinin-1 receptor antagonist; S-DEX with M-5HT, single dose of dexamethasone in combination with multiple doses of a first-generation 5-hydroxytryptamine3 receptor antagonist; S-DEX with M-5HT/NK1, single dose of dexamethasone in combination with multiple doses of a first-generation 5-hydroxytryptamine3 receptor antagonist and neurokinin-1 receptor antagonist; S-DEX with PALO, single dose of dexamethasone in combination with palonosetron; S-DEX with PALO/NK1, single dose of dexamethasone in combination with palonosetron and neurokinin-1 receptor antagonist; S-DEX with S-5HT/NK1, single dose of dexamethasone in combination with a single dose of a first-generation 5-hydroxytryptamine3 receptor antagonist and neurokinin-1 receptor antagonist.

Supplementary Table 4: Antiemetic regimens each study included in network meta-analysis

Study	Publication date	Anti-emetic regimen in Experimental arm
Aapro et al	2014	Dexamethasone (20mg qD) + Palonosetron (0.50mg qD)
		Dexamethasone (12mg qD) + Palonosetron (0.50mg qD) + Netupitant (300mg qD)
Celio et al[*3]	2012	Dexamethasone (8mg IV)+Palonosetron (0.25mg IV)
		Dexamethasone (8mg IV-8mg qD-8mg qD)+Palonosetron (0.25mg IV)
Herrstedt et al	2009	Dexamethasone (8mg)+Ondansetron (8mg BID-8mg BID-8mg BID)
		Dexamethasone (8mg IV)+Ondansetron (8mg BID-8mg BID-8mg BID)+Casopitant (150mg qD)
		Dexamethasone (8mg IV)+Ondansetron (8mg BID-8mg BID-8mg BID)+Casopitant (150mg qD-50mg qD-50mg qD)
		Dexamethasone (8mg IV)+Ondansetron (8mg BID-8mg BID-8mg BID)+Casopitant (90mg IV-50mg qD-50mg qD)
Ito et al	2018	Dexamethasone (12mg IV)+Palonosetron (0.75mg IV)+Aprepitant (125mg qD-80mg qD-80mg qD)/Fosaprepitant (150mg IV)
		Dexamethasone (12mg IV-8mg qD-8mg qD)+Palonosetron (0.75mg IV)+Aprepitant (125mg qD-80mg qD-80mg qD)/Fosaprepitant (150mg IV)
Kosaka et al	2016	Dexamethasone (12mg IV)+Palonosetron (0.75mg IV)+Aprepitant (125mg qD-80mg qD-80mg qD)
		Dexamethasone (12mg IV-8mg IV-8mg IV)+Palonosetron (0.75mg IV)+Aprepitant (125mg qD-80mg qD-80mg qD)
Matsumoto et al	2020	Dexamethasone (12-8-8)+Granisetron (1mg)+Fosaprepitant (150mg IV)
		Dexamethasone (12-8-8)+Palonosetron (0.75mg)+Fosaprepitant (150mg IV)

Ogata	2017	Dexamethasone (9.9mg IV)+Granisetron (40μg/kg IV) +Aprepitant (125mg qD-80mg qD-80mg qD)
		Dexamethasone (9.9mg IV)+Palonosetron (0.75mg IV) +Aprepitant (125mg qD-80mg qD-80mg qD)
Ohzawa et al	2015	Dexamethasone (13.2mg IV-8mg qD-8mg qD-8mg qD)+Granisetron (3mg IV)+Aprepitant (125mg qD-80mg qD-80mg qD)
		Dexamethasone (13.2mg IV-8mg qD-8mg qD-8mg qD)+Palo (0.75mg IV)+Aprepitant (125mg qD-80mg qD-80mg qD)
Rapoport et al	2010	Dexamethasone (20mg qD)+Ondansetron (8mg BID-8mg BID-8mg BID)
		Dexamethasone (12mg qD)+Ondansetron (8mg BID)+Aprepitant (125mg qD-80mg qD-80mg qD)
Roila et al	2014	Dexamethasone (8mg IV)+Palonosetron (0.25mg IV)+Aprepitant (125mg qD-80mg qD-80mg qD)
		Dexamethasone (8mg IV-8mg qD-8mg qD)+Palonosetron (0.25mg IV)+Aprepitant (125mg qD)
Saito et al	2009	Dexamethasone (16mg IV-4mg qD-4mg qD)+Granisetron (40μg/kg IV)
		Dexamethasone (16mg IV-4mg qD-4mg qD)+Palo (0.75mg IV)
Schnadig et al*3	2017	Dexamethasone (12mg IV-8mg qD-8mg BID-8mg BID)+Ondansetron (0.15mg/kg IV)+Fosaprepitant (150mg IV)
		Dexamethasone (12mg IV-8mg qD-8mg BID-8mg BID)+AFP530 (500mg SC)+Fosaprepitant (150mg IV)
Schwartzberg et al	2015	Dexamethasone (20mg qD)+Granisetron (2mg qD-2mg qD-2mg qD)
		Dexamethasone (20mg qD)+Granisetron (2mg qD-2mg qD-2mg qD)+Rolapitant (180 qD)
Schwartzberg et al	2020	Dexamethasone (12mg qD)+Palonosetron (0.5mg IV)+Netupitant (235mg IV)
		Dexamethasone (12mg qD)+Palonosetron (0.5mg qD)+Netupitant (300mg qD)
Segawa et al	2009	Dexamethasone (8mg IV)+Palonosetron (0.075mg IV)
		Dexamethasone (8mg IV)+Palonosetron (0.25mg IV)
		Dexamethasone (8mg IV)+Palonosetron (0.75mg IV)
Warr et al	2005	DEX (20mg qD)+OND (8mg BID-8mg BID-8mg BID)

		DEX (12mg qD)+OND (8mg BID)+Aprepitant (125mg qD-80mg qD-80mg qD)
Wenzell et al	2013	DEX (12mg qD-8mg qD-8mg qD-8mg qD)+OND (24mg qD)+Aprepitant (125mg qD-80mg qD-80mg qD)
		DEX (12mg qD-8mg qD-8mg qD-8mg qD)+Palo (0.25mg IV)+Aprepitant (125mg qD-80mg qD-80mg qD)
Yeo et al	2009	DEX (20mg qD)+OND (8mg BID-8mg BID-8mg BID)
		DEX (12mg qD)+OND (8mg BID)+Aprepitant (125mg qD-80mg qD-80mg qD)
Zelek et al	2021	DEX (8mg qD-8mg qD-8mg qD-8mg qD)+Palonosetron (0.5mg qD)+Netupitant (300mg qD)
		DEX (8mg qD-8mg qD-8mg qD-8mg qD)+Ondansetron (8mg IV)+Aprepitant (125mg qD-80mg qD-80mg qD)

Supplementary Figure 1: Risk of bias summary: review authors' judgements about each risk of bias item for each included study.

Study ID	D1	D2	D3	D4	D5	Overall
Aapro_2014	+	+	+	+	+	+
Celio_2012	+	+	+	!	+	!
Herrstedt_2009	+	+	+	+	+	+
Ito_2018	+	+	+	+	+	+
Kosaka_2016	+	+	+	+	+	+
Matsumoto_2020	+	!	+	+	+	!
Ogata_2017	+	!	+	+	+	!
Ohzawa_2015	+	+	+	!	+	!
Rapoport_2010	+	!	+	+	+	!
Roila_2014	+	+	+	+	+	+
Saito_2009	+	+	+	+	+	+
Schnadig_2017	+	!	+	+	+	!
Schwartzberg_2020	+	+	+	+	+	+
Schwartzberg_2015	+	+	+	+	+	+
Segawa_2009	+	+	+	+	+	+
Warr_2005	+	+	+	+	+	+
Wenzell_2013	+	+	+	!	+	!
Yeo_2009	+	+	+	+	+	+
Zelek_2021	+	+	+	!	+	!

+ Low risk
! Some concerns
- High risk

D1 Randomisation pr
D2 Deviations from tl
D3 Missing outcome
D4 Measurement of t
D5 Selection of the re

Supplementary Figure 2: Risk of bias graph

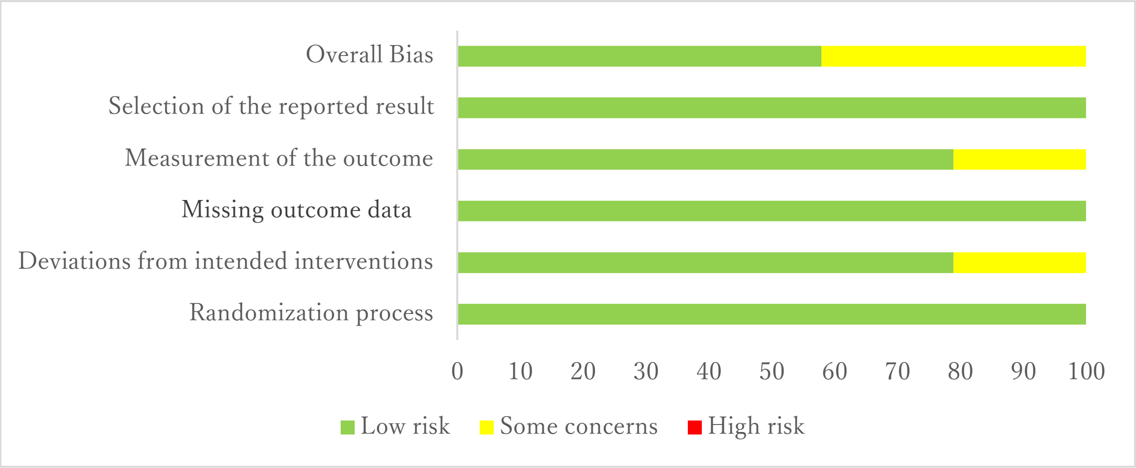

Supplementary Table 5: Proportion of patients achieving a complete response during the delayed phase in eligible studies

Study (PMID)	Acronym	Complete response rete during delayed phase (%)
Aapro 2014	S-DEX with PALO	69.5 (504/725)
(24603643)	S-DEX with PALO/NK1	76.9 (557/724)
Celio 2011	S-DEX with PALO	55.8 (29/52)
(20574663)	M-DEX with PALO	75.4 (46/61)
Herrstedt 2009	S-DEX with M-5HT	58.9 (282/479)
(19805683)	S-DEX with M-5HT/NK1	72.9 (349/479)
	S-DEX with M-5HT/NK1	72.9 (350/480)
	S-DEX with M-5HT/NK1	73.9 (354/479)
Ito 2018	S-DEX with PALO/NK1	49.7 (77/155)
(29443652)	M-DEX with PALO/NK1	53.0 (80/151)
Kosaka 2016	S-DEX with PALO/NK1	94.9 (37/39)
(26349772)	M-DEX with PALO/NK1	87.8 (36/41)
Matsumoto 2020	M-DEX with S-5HT/NK1	60.4 (99/164)
(32168551)	M-DEX with PALO/NK1	62.3 (101/162)
Ogata 2017	S-DEX with S-5HT/NK1	53.9 (132/245)
(Not applicable)	S-DEX with PALO/NK1	58.5 (144/246)
Ohzawa 2015	M-DEX with S-5HT/NK1	71.4 (15/21)
(25435944)	M-DEX with PALO/NK1	73.7 (14/19)
Rapoport 2020	S-DEX with M-5HT	52.9 (108/204)
(19568773)	S-DEX with S-5HT/NK1	64.8 (129/199)
Roila 2014	S-DEX with PALO/NK1	79.5 (221/278)
(24323030)	M-DEX with PALO/NK1	79.5 (217/273)
Saito 2009	M-DEX with S-5HT	50.0 (118/236)
(19135415)	M-DEX with PALO	61.1 (146/239)
Schnadig 2017	M-DEX with S-5HT/NK1	56.0 (167/298)
(28579832)	M-DEX with M-5HT/NK1	63.6 (185/291)
Schwartzberg 2015	S-DEX with M-5HT	59.6 (214/359)
(26272768)	S-DEX with M-5HT/NK1	66.9 (230/344)

Schwartzberg 2020 (32162813)	S-DEX with PALO/NK1	75.5 (151/200)
	S-DEX with PALO/NK1	78.7 (159/202)
Segawa 2009 (19605507)	S-DEX with PALO	38.5 (10/26)
	S-DEX with PALO	48.1 (13/27)
	S-DEX with PALO	63.0 (17/27)
Warr 2005 (15837996)	S-DEX with M-5HT	49.1 (208/424)
	S-DEX with S-5HT/NK1	55.0 (238/433)
Wenzell 2013 (23748485)	M-DEX with S-5HT/NK1	45.0 (9/20)
	M-DEX with PALO/NK1	65.0 (13/20)
Yeo 2009 (18327706)	S-DEX with M-5HT	58.1 (36/62)
	S-DEX with S-5HT/NK1	64.5 (40/62)
Zelek 2021 (34216177)	M-DEX with PALO/NK1	89.7 (70/78)
	M-DEX with S-5HT/NK1	86.9 (73/84)
Matsuura 2022 (35045185)	S-DEX with PALO/NK1	Not applicable
	S-DEX with PALO/NK1	Not applicable
Herrstedt 2005 (16104039)	S-DEX with M-5HT	Not applicable
	S-DEX with S-5HT/NK1	Not applicable

Supplementary Table 6: Pooled proportion of patients achieving a complete response during the delayed phase for each antiemetic regimen using random- and fixed-effect models

Antiemetic regimen			Cohort using the regimen (number of studies)	Complete response during delayed phase /patients using regimen	Pooled proportion of complete response, random effect (95% CI)	Pooled proportion of complete response, fixed effect (95% CI)	Heterogeneity I^2 statistics
DEX dose	5HT3 RA	NK1 RA					
Multiple	PALO	Use	7 (7 study)	531/744	74.4% (61.9–83.8)	69.4% (65.8–72.8)	88.9%
Single	PALO	Use	7 (6 study)	1346/1844	73.9% (62.5–82.8)	72.1% (69.9–74.2)	93.1%
Multiple	Single dose of 1st 5HT3 RA	Use	5 (5 study)	363/587	65.6% (49.1–79.1)	60.4% (56.2–64.4)	84.6%
Single	Single dose of 1st 5HT3 RA	Use	4 (4 study)	539/939	58.6% (52.7–64.3)	57.3% (54.1–60.5)	62.5%
Multiple	Multiple doses of 1st 5HT3 RA	Use	1 (1 study)	185/291	NA	NA	NA
Single	Multiple doses of 1st 5HT3 RA	Use	4 (2 study)	1283/1782	71.8% (68.9–74.6)	71.9% (69.8–74.0)	47.5%

Multiple	PALO	Absent	2 (2 study)	192/300	67.5% (52.1–79.9)	63.7% (58.1–69.0)	76.4%
Single	PALO	Absent	5 (3 study)	573/857	57.2% (45.5–68.1)	66.8% (63.5–69.9)	77.6%
Multiple	Single dose of 1st 5HT3 RA	Absent	1 (1 study)	118/236	NA	NA	NA
Single	Multiple doses of 1st 5HT3 RA	Absent	5 (5 study)	848/1528	55.5% (50.9–60.0)	55.5% (52.9–58.0)	67.9%
